# Population-Level Frequency of Fluoroquinolone Resistance by Whole-Genome Sequencing Drug Predictions in *Mycobacterium tuberculosis* Complex Isolates in England From 2017 Through 2023

**DOI:** 10.1093/cid/ciae560

**Published:** 2024-11-13

**Authors:** Elena Ferran, Cathleen Chan, Noorann Sheikh, Martin Dedicoat, Eliza Alexander, Ana Gibertoni-Cruz, James Brown, Esther Robinson, Marc Lipman

**Affiliations:** National Mycobacterial Reference Service, UK Health Security Agency, Birmingham, United Kingdom; Respiratory Medicine, Royal Free London NHS Foundation Trust, London, United Kingdom; National Mycobacterial Reference Service, UK Health Security Agency, Birmingham, United Kingdom; National Mycobacterial Reference Service, UK Health Security Agency, Birmingham, United Kingdom; National Mycobacterial Reference Service, UK Health Security Agency, Birmingham, United Kingdom; Department of Infectious Diseases, University Hospitals Birmingham NHS Foundation Trust, Birmingham, United Kingdom; National Mycobacterial Reference Service, UK Health Security Agency, Birmingham, United Kingdom; National Mycobacterial Reference Service, UK Health Security Agency, Birmingham, United Kingdom; Respiratory Medicine, Royal Free London NHS Foundation Trust, London, United Kingdom; Division of Medicine, University College London, London, United Kingdom; National Mycobacterial Reference Service, UK Health Security Agency, Birmingham, United Kingdom; Respiratory Medicine, Royal Free London NHS Foundation Trust, London, United Kingdom; Division of Medicine, University College London, London, United Kingdom

**Keywords:** *Mycobacterium tuberculosis*, fluoroquinolone resistance, whole-genome sequencing, drug therapy

## Abstract

Fluoroquinolones are increasingly important for anti-tuberculosis (TB) treatment. Identifying fluoroquinolone resistance (FR) is essential. Using sequencing data from over 16 000 unselected isolates, this first English survey of FR, found it present in 1.4% overall and 23.9% of multidrug-resistant TB. Routine sequencing allows clinically-relevant resistance surveillance and should be widely adopted.

Recent tuberculosis (TB) trial data have demonstrated that both drug-sensitive (DS), defined as a strain of *Mycobacterium tuberculosis* sensitive to standard first-line agents (rifampicin, isoniazid, pyrazinamide, and ethambutol), and drug-resistant (DR) TB can be treated effectively with short-course fluoroquinolone-containing combination therapy. Current World Health Organization (WHO) recommendations include use of a 4-month treatment regimen that contains a fluoroquinolone throughout for DS-TB and 6-month fluoroquinolone-containing regimens for DR-TB, including rifampicin-resistant (RR) TB and multidrug-resistant (MDR) TB [1–3].

The adverse event profile of fluoroquinolones is well recognized; however, less is known about the prevalence of fluoroquinolone resistance, especially in DS-TB. This is a prerequisite if the new regimens are to be prescribed appropriately. WHO reported that global coverage of testing for resistance to fluoroquinolones was 50% in 2021, although the rates of fluoroquinolone resistance are less clear [4]. A multicountry surveillance study from 2016 that used phenotypic drug susceptibility testing (DST) showed that fluoroquinolone resistance varied from 0.5% of isolates in South Africa to 10.7% of isolates in Pakistan. Overall, there was less fluoroquinolone resistance than rifampicin resistance other than in Pakistan and Bangladesh. In all countries, it was found that fluoroquinolone resistance was higher in people with RR-TB compared with those with DS-TB [5].

Since 2017, whole-genome sequencing (WGS) on all *M. tuberculosis* complex (MTBC) isolates in England by the UK Health Security Agency (UKHSA) National Mycobacterial Reference Service (NMRS) has been routine. This sequencing provides species identification, relatedness, and drug susceptibility predictions for all first-line agents, fluoroquinolones, and aminoglycosides in 7 working days [6].

Here, we report the first retrospective study to use routine WGS drug-resistance predictions to fluoroquinolones to assess the population-level frequency of fluoroquinolone resistance in contemporary clinical *M. tuberculosis* complex isolates in England.

## METHODS

Culture-positive isolates were sequenced and processed by UKHSA at the NMRS laboratories. The sample processing procedure was as previously described [7]. Acceptance criteria for reporting included mapping to positive control and reference strain (H37Rv), with mapped coverage at >80% and a median depth of >10 using *Mykrobe*. The number of reads mapped must be >0.50 million bases [8]. Data for all *M. tuberculosis* complex isolates sequenced between January 2017 and March 2023 are included in this study. Data were cleaned to include only 1 sample per patient, and quality assurance samples were excluded. All statistical analysis was carried out independently by 2 authors (E. F. and C. C.) to ensure concordance of results.

The WGS service at NMRS uses the COMPASS (COMplete Pathogen Analytical Software Solution) bioinformatics pipeline [7]. This uses a custom mutation catalog that incorporates data informed by Walker et al, as previously described [8, 9].

WGS drug predictions by NMRS produced 1 of 4 outcomes: *resistant*: where a known resistance-conferring mutation to a drug has been detected; *sensitive*: indicates that no mutation associated with resistance or nonsynonymous mutation in a gene of interest has been detected; *unknown*: a nonsynonymous mutation in a gene of interest is identified, but data are lacking to reliably predict if sensitive or resistant (uncharacterized), these isolates often go on to have phenotypic testing for that drug; and *failed*: prediction can be due to a technical fail, for example, if there were a problem with the WGS process, insufficient mycobacteria in the positive culture, contamination with another organism, or there is a mixed mycobacterial population.

WHO now stratifies mutations into 1 of 5 categories: associated with resistance; associated with resistance-interim; uncertain significance; not associated with resistance-interim; and not associated with resistance [10]. COMPASS has not yet been updated along with the WHO catalog of mutations in *M. tuberculosis* complex and their association with drug resistance. Consequently, there are some mutations that differ in interpretation when automatically generated compared with the WHO interpretation. At NMRS, these undergo amendments at the reporting stage before results are released to service users.

COMPASS WGS prediction for fluoroquinolone refers to the fluoroquinolone group of drugs and does not subspecify for individual fluoroquinolones. However, information on specific mutations is used to provide an interpretative report to service users.

Our analysis focused on the relationships among fluoroquinolone, isoniazid, and rifampicin drug susceptibility predictions by WGS as well as resistance over time.

## RESULTS

### Species Identified

A total of 16 874 isolates were successfully sequenced for species identification: 16 185 (95.9%) were *M. tuberculosis*, 217 (1.3%) were Bacillus Calmette-Guérin (BCG), 159 (0.9%) were *Mycobacterium bovis*, 194 (1.1%) were *Mycobacterium africanum*, 5 (0.0%) were *Mycobacterium Microti*, and 114 (0.7%) could only be speciated to the *M. tuberculosis* complex level.

### Frequency of Overall Anti-TB Drug Resistance as Predicted by WGS

Because 7 isolates were mixed with non-tuberculous mycobacteria species, drug susceptibility predictions were not obtainable. Table 1 shows the genotypic predictions for each drug for 16 867 MTBC isolates. A total of 1.4% (n = 228 of 16 867) were predicted to be resistant to fluoroquinolones; this is less than the frequency of resistance predicted for the first-line agents. There was a lower frequency of isolates predicted to be sensitive to fluoroquinolone (84.4%, n = 14 234 of 16 867) compared with the first-line agents. A generally greater proportion of isolates had mutations of unknown significance for fluoroquinolones (12.3%, n = 2081 of 16 867) and ethambutol (12.5%, n = 2102 of 16 867).

**Table 1.** Frequency (Number and Percentage) of Whole-Genome Sequencing (WGS)–Predicted Sensitive, Resistant, and Unknown Mutations and Failed Predictions in *Mycobacterium tuberculosis* Complex Isolates Sequenced Using WGS at the National Mycobacterial Reference Service: 2017–2023

WGS drug predictions	Isoniazid	Rifampicin	Ethambutol	Pyrazinamide	Quinolones
Sensitive	14 701 (87.2)	15 026 (89.1)	14 334 (85.0)	15 557 (92.2)	14 234 (84.4)
Resistant	1277 (7.6)	400 (2.4)	343 (2.0)	556 (3.3)	228 (1.4)
Unknown mutation	644 (3.8)	464 (2.8)	2102 (12.5)	224 (1.3)	2081 (12.3)
Failed	245 (1.5)	977 (5.8)	88 (0.5)	530 (3.1)	324 (1.9)
**Total**	**16 867**	**16 867**	**16 867**	**16 867**	**16 867**

### Frequency of Fluoroquinolone Resistance in MTBC With Different Drug-Resistance Patterns

Fluoroquinolone resistance was present in 0.8% of isolates with DS TB (n = 91 of 11 198), 1.1% of isolates with isoniazid monoresistance (n = 8 of 711), 4.2% of isolates with RR TB (n = 3 of 72), and 23.9% of MDR-TB isolates with resistance to both rifampicin and isoniazid (n = 73 of 306). Supplementary Table 1 shows all data for the combinations with fluoroquinolone predictions.

### Fluoroquinolone Resistance Over Time

**Figure 1.**
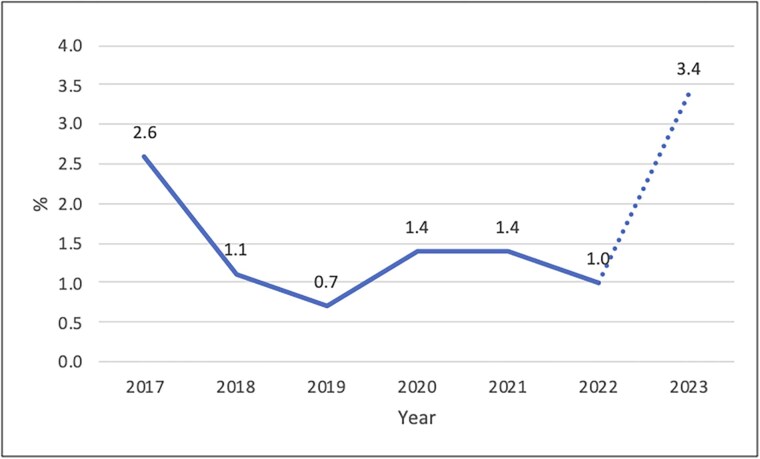
Rates of fluoroquinolone resistance predicted by whole-genome sequencing in all tested *Mycobacterium tuberculosis* complex isolates in England from January 2017 to March 2023.

The annual frequency of fluoroquinolone resistance ranged from 0.7% in 2019 to 3.4% in January 2023–March 2023. Figure 1 shows the annual frequency of fluoroquinolone resistance over time.

## DISCUSSION

The routine use of WGS on all *M. tuberculosis* complex isolates in England by NMRS enabled analysis of DR TB rates. Our data show that fluoroquinolone-resistant *M. tuberculosis* is present at a low though detectable frequency in DS (0.8%) and isoniazid-resistant (1.1%) TB isolates in England. However, fluoroquinolone resistance increases with the presence of rifampicin resistance (4.2%) and MDR-TB (23.9%).

The level of fluoroquinolone resistance is similar to that previously reported in other countries, although differences between study data methods limit direct comparison [5]. We used predictions by WGS to identify resistance to the fluoroquinolone group overall, whereas previous studies have used phenotypic drug susceptibility testing (pDST) for different fluoroquinolones at different concentrations.

In 12.3% of MTBC isolates, an unknown (uncharacterized) mutation was detected, generating an “unknown” prediction for fluoroquinolone susceptibility. A total of 1021 of these isolates went on to have pDST, and only 0.4% were confirmed to be quinolone-resistant. A change to the current bioinformatics pipeline used by NMRS to incorporate the most recent and updated WHO catalog of resistant mutations in *M. tuberculosis* complex will likely reduce this proportion [10]. However, where fluoroquinolone susceptibility information is currently required, further pDST is performed at NMRS, leading to additional time to a result, greater use of resources, and higher costs.

Determining fluoroquinolone susceptibility is of increasing importance. For the treatment of both DS TB and DR TB, WHO advocates regimens where a fluoroquinolone is central to treatment success. Furthermore, a fluoroquinolone is sometimes substituted in regimens where first-line agents are problematic due to drug toxicity. Therefore, providing this drug susceptibility prediction enables safe and effective treatment implementation.

Over time, the rates of quinolone resistance have remained between 0.7% and 2.6% (Figure 1). Our data showed an increase to 3.4% over the first 3 months of 2023. While these are partial data for the year and therefore difficult to extrapolate, this may be real as there was an increase in the incidence rate and absolute number of MDR-TB diagnoses in England between 2022 and 2023 (with 43 cases in 2022 and 72 cases in 2023) [11].

WHO annual TB reports do not currently include information on fluoroquinolone resistance, and reports from UKHSA only do so for MDR strains. We have shown that this information is readily available from routine WGS to guide treatment decisions early in management and improve surveillance for drug resistance. We hope that our data for England will soon be reproduced internationally and provide an accurate global picture of fluoroquinolone resistance.

## CONCLUSIONS

Overall fluoroquinolone resistance in *M. tuberculosis* in England is low at 1.4%. The frequency of fluoroquinolone resistance increases with other drug resistance to almost 24% in MDR-TB. Knowing whether or not a mycobacterial isolate is fluoroquinolone-susceptible is vital, given the drug's central role in WHO-recommended regimens for all TB cases. Detection, reporting, and monitoring of fluoroquinolone resistance can be performed where routine sequencing of TB is available and should be established globally as sequencing methods are further introduced worldwide.

## Supplementary Data

Supplementary materials are available at *Clinical Infectious Diseases* online. Consisting of data provided by the authors to benefit the reader, the posted materials are not copyedited and are the sole responsibility of the authors, so questions or comments should be addressed to the corresponding author.

## Supplementary Material

ciae560_Supplementary_Data

## Notes

***Author Contributions*.** Conception: M. L., E. R. Data collection: routine whole-genome sequencing data: UK Health Security Agency (UKHSA) National Mycobacterial Reference Service (NMRS)) as part of clinical care in England. Data analysis: E. F., C. C. Drafting of the manuscript: E. F. (first draft), C. C. (reviewed edits from all authors). Review of the manuscript: All authors. Final approval: M. L., E. R.

***Acknowledgment.*** The authors thank everyone at the UKHSA NMRS.
