# Supplementary material for: Population-Level Frequency of Fluoroquinolone Resistance by Whole-Genome Sequencing Drug Predictions in Mycobacterium tuberculosis Complex Isolates in England From 2017 Through 2023
Source: Clin Infect Dis. 2024 Nov 13;80(3):660–2. doi: 10.1093/cid/ciae560 (PMC11912959; doi:10.1093/cid/ciae560)
Supplement: ciae560_Supplementary_Data [file ciae560_supplementary_data.docx]

Supplementary data

**Supplementary Table 1:** Frequency of fluoroquinolone resistance predictions by WGS in *M. tuberculosis* complex (MTBC) with different drug resistance patterns in England from 2017-2023

|  |  | Quinolone prediction (n, %) | | | |
| --- | --- | --- | --- | --- | --- |
| WGS drug prediction combination | Number | Resistant | Sensitive | Unknown | Failed |
| DS TB (RHZE all = S) | 11198 | 91 (0.8) | 9753 (87.1) | 1312 (11.7) | 42 (0.4) |
| DS TB (H = S, RZE = S or U) | 13234 | 107 (0.8) | 11429 (86.4) | 1651 (12.5) | 47 (0.4) |
| H mono-res TB (H = R, RZE = S) | 711 | 8 (1.1) | 628 (88.3) | 71 (10.0) | 4 (0.6) |
| H mono-res TB (H = R, RZE = S or U) | 807 | 9 (1.1) | 707 (87.6) | 87 (10.8) | 4 (0.5) |
| RIF mono-res TB (R = R, HZE = S) | 72 | 3 (4.2) | 67 (93.1) | 2 (2.8) | 0 (0.0) |
| RIF mono-res TB (R = R, H = S, ZE = S or U) | 73 | 3 (4.1) | 68 (93.2) | 2 (2.7) | 0 (0.0) |
| RIF mono-res TB (R = R, HZE = S or U) | 77 | 4 (5.2) | 70 (90.9) | 3 (3.9) | 0 (0.0) |
| MDR-TB (RH = R, ZE = S or U or R) | 268 | 62 (23.1) | 175 (65.3) | 30 (11.2) | 1 (0.4) |
| MDR-TB (RH = R) | 306 | 73 (23.9) | 197 (64.4) | 34 (11.1) | 2 (0.7) |
| Drugs: R/RIF = rifampicin, H = isoniazid, Z = pyrazinamide, E = ethambutol  Prediction: R = resistant, S = sensitive, U = unknown, F = failed  DS = drug-sensitive, MDR = multi-drug resistant  Mono-res = mono-resistant | | | | | |
